# Quantitative Flow Ratio-Guided vs. Angiography-Guided Percutaneous Coronary Intervention: A Systematic Review and Meta-Analysis of One-Year Clinical Outcomes †

**DOI:** 10.3390/jcm14145015

**Published:** 2025-07-15

**Authors:** Viet Nghi Tran, Amreen Dhindsa, Kuchalambal Agadi, Hoang Nhat Pham, Hong Hieu Truong, Chau Doan Nguyen, Hanad Bashir, Huan Dat Pham, Thanh Vien Truong, Phillip Tran, Thach Nguyen

**Affiliations:** 1 Department of Internal Medicine, Weiss Memorial Hospital, Chicago, IL 60640, USA; dramreendhindsa@gmail.com (A.D.); kuchalambal@gmail.com (K.A.); 2 Department of Internal Medicine, The University of Arizona, Tucson, AZ 85721, USA; npham917@arizona.edu; 3 Department of Internal Medicine, Ascension Saint Francis Hospital, Evanston, IL 60202, USA; hhieu.truong@gmail.com; 4 Department of Internal Medicine, Texas Tech University Health Science Center, Amarillo, TX 79106, USA; doannguyenmd92@gmail.com; 5 Department of Cardiology, The Christ Hospital Heart and Vascular Institute, Cincinnati, OH 45219, USA; hanad.bashir@thechristhospital.com (H.B.); thanhvientruong1988@gmail.com (T.V.T.); 6 The Lindner Center for Research and Education, Cincinnati, OH 45219, USA; 7 Department of Internal Medicine, Conemaugh Memorial Medical Center, Johnstown, PA 15905, USA; huandat123@gmail.com; 8 Department of Internal Medicine, Nam Can Tho University, Can Tho 900000, Vietnam; dr.philtran@gmail.com; 9 Department of Cardiology, Methodist Hospital, La Porte, IN 46350, USA; thachnguyen2000@yahoo.com

**Keywords:** MACE, QFR-guided PCI, quantitative flow ratio (QFR), percutaneous coronary intervention (PCI), revascularization, FFR, iFR, vFFR

## Abstract

**Background:** Quantitative Flow Ratio (QFR) is a novel, wire-free, and hyperemia-free physiological assessment for guiding Percutaneous Coronary Intervention (PCI), which may offer advantages over traditional angiography-guided PCI. This systematic review with meta-analysis compares clinical outcomes after one year in patients who underwent QFR-guided versus angiography-guided PCI. **Methods:** This study was conducted following the Preferred Reporting Items for Systematic Reviews and Meta-Analyses (PRISMA) guidelines and was registered on 4 November 2024 in PROSPERO (ID: CRD42024609799). A systematic search was performed across multiple databases to identify clinical trials comparing QFR-guided and angiography-guided PCI. Random-effects models were used to assess one-year outcomes of major adverse cardiovascular events (MACEs), revascularization, and rehospitalization, with heterogeneity measured using I^2^, H^2^, and Cochran’s Q statistics. Study quality was evaluated using the Cochrane Risk of Bias 2 (RoB 2) tool. **Results:** Compared to traditional angiography-guided PCI, QFR-guided PCI was associated with numerically lower but statistically non-significant risks of MACEs (risk difference: −0.08, 95% CI: −0.20 to 0.04), revascularization (risk difference: −0.02, 95% CI: −0.08 to 0.03), and rehospitalization (risk difference: −0.02, 95% CI: −0.08 to 0.04) over one year. Substantial heterogeneity was observed for MACEs (I^2^ = 84.95%, H^2^ = 6.64) and revascularization (I^2^ = 94.18%, H^2^ = 17.18), whereas rehospitalization exhibited low heterogeneity (I^2^ = 17.17%, H^2^ = 1.21). The risk of bias was assessed by the RoB 2 tool, which revealed low to some concern risk of bias across key domains. **Conclusions:** Quantitative Flow Ratio (QFR) has demonstrated comparable one-year clinical outcomes to traditional angiography for PCI guidance, with a trend toward improved results. However, the high heterogeneity among studies and the risk of bias necessitate the need for larger, high-quality trials to validate these findings.

## 1. Introduction

Percutaneous Coronary Intervention (PCI) plays a central role in the management of coronary artery disease (CAD), particularly in patients with obstructive atherosclerosis. Traditionally, PCI decisions have relied on angiographic imaging; however, this anatomical assessment can misrepresent lesion severity, sometimes resulting in unnecessary or inadequate interventions. To address this limitation, Fractional Flow Reserve (FFR) was introduced as a physiological method for assessing coronary stenoses, utilizing a pressure wire and pharmacologic hyperemia to measure their functional significance. Clinical trials have consistently shown that FFR-guided PCI improves patient outcomes and reduces the rate of unwarranted procedures [1,2]. Despite its clinical benefits, the use of FFR in routine practice remains limited due to its technical complexity, the need for vasodilators like adenosine, and the associated patient discomfort [3]. Additionally, results may be influenced by factors such as microvascular dysfunction and caffeine intake [4,5]. In response, the Instantaneous Wave-Free Ratio (iFR) was developed to provide a simpler alternative. Unlike FFR, iFR does not require pharmacologic hyperemia, making it faster, more comfortable, and better tolerated, particularly in patients with contraindications to vasodilators. Large-scale trials such as DEFINE-FLAIR and iFR-SWEDEHEART have demonstrated that iFR is non-inferior to FFR for guiding coronary revascularization [6,7].

More recently, the Quantitative Flow Ratio (QFR) has emerged as a novel, image-based method for physiological assessment [8]. QFR offers several advantages over both iFR and FFR by eliminating the need for a pressure wire or hyperemic agents, while maintaining diagnostic accuracy comparable to both modalities [9,10,11,12,13]. Derived from standard coronary angiography, QFR is wire-free, hyperemia-free, reduces procedural time, minimizes patient discomfort, and carries no pharmacologic risk. It can also be applied retrospectively, enabling functional evaluation without additional procedural steps [14]. These features make QFR a promising tool for streamlining PCI workflows and expanding access to physiology-guided decision-making. However, whether QFR-guided PCI translates into better clinical outcomes than conventional angiography-guided PCI remains uncertain. Several randomized trials have sought to evaluate this question, but their findings have been inconsistent [15,16,17]. To address this gap, we conducted a systematic review and meta-analysis of randomized controlled trials comparing QFR-guided versus angiography-guided PCI. Our objective was to assess one-year clinical outcomes, including major adverse cardiovascular events (MACEs), repeat revascularization, and rehospitalization. This study aims to clarify the clinical utility of QFR and inform future strategies for physiologically guided PCI. This article is a revised and expanded version of the abstract entitled “Quantitative Flow Ratio (QFR)-Guided Vs. Angiography-Guided Percutaneous Coronary Intervention (PCI): A Meta-Analysis of One-Year Clinical Outcomes”, which was presented at the American College of Cardiology conference on 29 March 2025, in Chicago, USA [18].

## 2. Methods

### 2.1. Data Sources and Search Strategy

This study adhered to the Preferred Reporting Items for Systematic Reviews and Meta-Analyses (PRISMA) guidelines and was registered in PROSPERO (ID: CRD42024609799). We conducted a comprehensive search of five electronic databases: PubMed, ClinicalTrials.gov, Embase, Scopus, and the Cochrane CENTRAL database. The search included articles published from 1 January 2000 through 30 November 2024 and was restricted to English-language studies involving human subjects. Search terms included combinations of “QFR” OR “quantitative flow ratio,” “PCI” OR “percutaneous coronary intervention,” and outcomes-related terms such as “MACE,” “myocardial infarction,” “ischemic heart disease,” “revascularization,” “stroke,” or “mortality.” Boolean operators were applied to link these terms systematically as follows: (QFR OR quantitative flow ratio) AND (PCI OR percutaneous coronary intervention) AND (MACE OR myocardial infarction OR ischemic heart disease OR revascularization OR stroke OR mortality). Duplicate records were removed before screening.

### 2.2. Selection Criteria

Eligible studies met the following inclusion criteria: randomized controlled trials or clinical trials involving adult patients (age ≥ 18 years), comparing QFR-guided PCI with angiography-guided PCI, and reporting one-year follow-up outcomes related to MACEs, myocardial infarction, revascularization, rehospitalization, stroke, or mortality. Studies were excluded if they were in vitro or animal studies, lacked a control group, or were only available as abstracts without full text.

### 2.3. Data Extraction

Two independent reviewers screened titles and abstracts for relevance, followed by full-text review to determine eligibility. From each included study, data were extracted on authorship, year of publication, country, sample size, study design, baseline patient characteristics, intervention details, follow-up duration, and reported clinical outcomes. Disagreements during the review or extraction process were resolved through consensus or arbitration by a third reviewer.

### 2.4. Outcomes

The primary outcome was the incidence of major adverse cardiovascular events (MACEs) at one year. Secondary outcomes included rates of repeat revascularization and rehospitalization.

### 2.5. Quality Assessment

The risk of bias for each included study was assessed using the Cochrane Risk of Bias 2 (RoB 2) tool. This tool evaluates five domains: randomization process, deviations from intended interventions, missing outcome data, measurement of outcomes, and selection of reported results. Each domain was rated as low, high, or having some concerns regarding bias [19].

### 2.6. Statistical Analysis

Statistical analyses were performed using a random-effects model (DerSimonian–Laird method) to account for between-study variability. Pooled risk differences (RDs) with 95% confidence intervals (CIs) were calculated for each outcome. Heterogeneity was evaluated using Cochran’s Q, I^2^, and H^2^ statistics, with I^2^ values > 75% indicating substantial heterogeneity. Egger’s and Begg’s tests were planned to evaluate publication bias if more than 10 studies were included [20]. The *p*-values were 2-sided with *p* < 0.05 indicating statistically significant. All analyses were conducted using STATA version 17 [21].

## 3. Results

### 3.1. Study Selection and Characteristics

**Figure 1:**
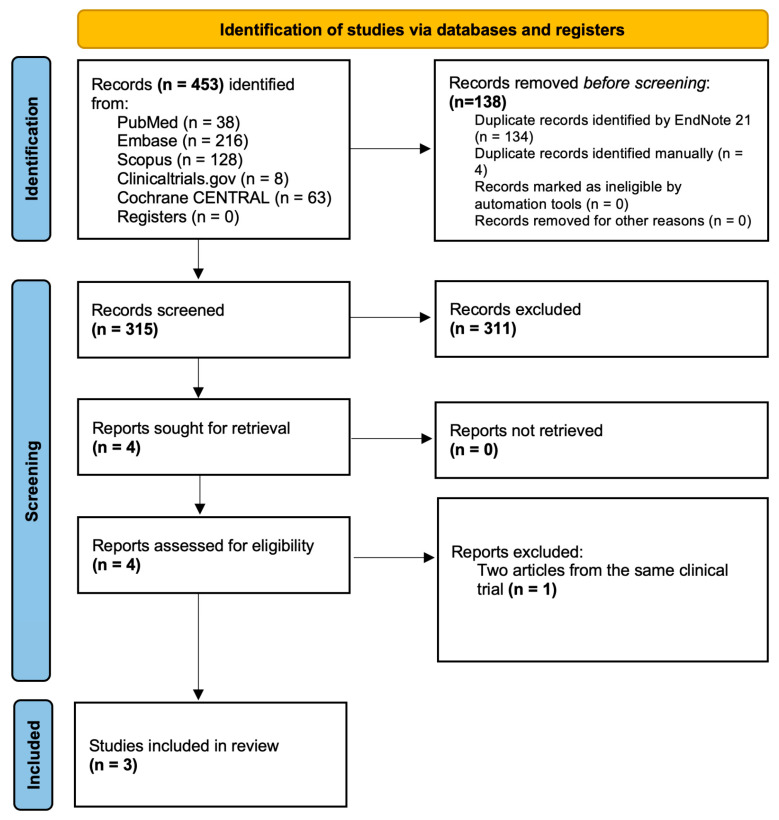
PRISMA flow diagram for results of the systematic search.

The initial database search identified 548 records. After the removal of duplicates and screening of titles and abstracts, four full-text articles were reviewed for eligibility. Ultimately, three studies met the inclusion criteria and were included in the meta-analysis (Figure 1). These studies collectively enrolled 4225 patients, with 2112 undergoing QFR-guided PCI and 2113 treated with angiography-guided PCI. All studies had a one-year follow-up period and reported data on MACEs and repeat revascularization. Two out of the three studies reported rates of rehospitalization in a one-year follow-up. Formal tests for publication bias were not performed because the results of common publication bias tests are not reliable with fewer than ten studies.

The included studies were conducted across different geographic regions and varied in sample size and population characteristics. However, they shared broadly similar designs, enrolling patients with obstructive coronary artery disease and using comparable definitions for clinical outcomes. Baseline demographics and procedural characteristics were well-balanced between treatment groups in all studies. Baseline characteristics, including definitions of MACEs in each study, are reported in Table 1.

### 3.2. Pooled Analysis

**Figure 2:**
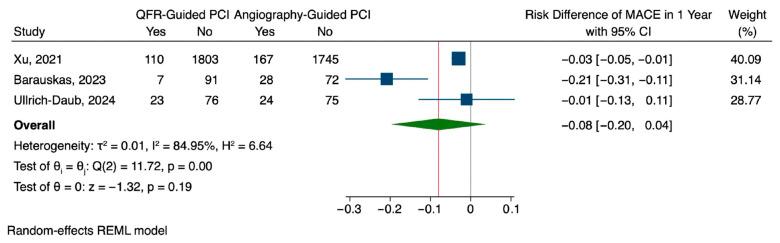
Pooled analysis for MACEs [15,16,17].

**Figure 3:**
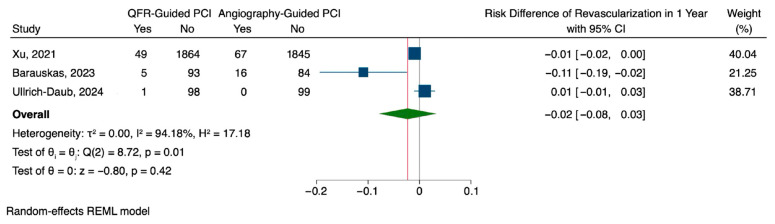
Pooled analysis for repeated revascularization [15,16,17].

**Figure 4:**
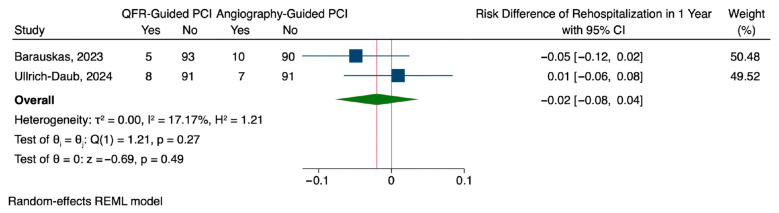
Pooled analysis for rehospitalization [15,16].

QFR-guided PCI was associated with a lower rate of major adverse cardiovascular events (MACEs) at one year compared to angiography-guided PCI, although the difference was not statistically significant. The pooled risk difference was −0.08 (95% CI: −0.20 to 0.04), with considerable heterogeneity observed across studies (I^2^ = 84.95%, H^2^ = 6.64) (Figure 2). Regarding repeat revascularization, QFR guidance showed a modest risk reduction of 0.02 compared to angiography-guided PCI (95% CI: −0.08 to 0.03), which was also not statistically significant. This outcome demonstrated substantial heterogeneity (I^2^ = 94.18%, H^2^ = 17.18), suggesting differences in treatment effect across studies (Figure 3). Rehospitalization rates followed a similar pattern, with a slight, non-significant reduction in the QFR group (risk difference: −0.02, 95% CI: −0.08 to 0.04). Unlike the other outcomes, the findings of rehospitalization were more consistent with low heterogeneity observed among studies (I^2^ = 17.17%, H^2^ = 1.21) (Figure 4).

### 3.3. Risk of Bias

**Figure 5:**
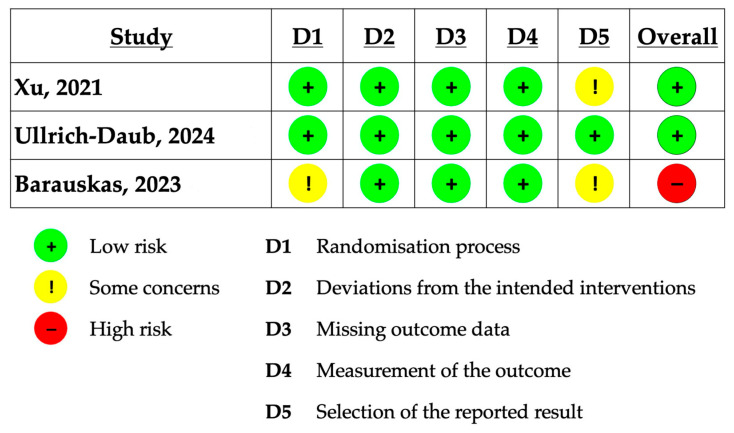
Cochrane Risk of Bias 2 Assessment [15,16,17].

A risk of bias assessment using the Cochrane Risk of Bias 2 (RoB 2) tool revealed low to some concerns across five domains in the included trials. The summary of quality assessment is reported in Figure 5. The main sources of bias included lack of blinding, potential deviations from intended interventions, and selective reporting of outcomes. These methodological limitations may have influenced the reliability of reported results and contributed to the observed heterogeneity.

## 4. Discussion

Ischemic heart disease (IHD) remains the leading cause of age-standardized Disability-Adjusted Life Years (DALYs) globally, contributing 2275.9 DALYs per 100,000 individuals [22]. Central to IHD is coronary artery disease, characterized by structural or functional abnormalities that impairs myocardial perfusion. Over the past two decades, the field of interventional cardiology has witnessed continuous innovation, ranging from timing and type of revascularization to advancements in antiplatelet therapy and physiologic lesion assessment. Despite these developments, angiography-guided percutaneous coronary intervention remains the most widely practiced approach. However, this traditional strategy is purely anatomical and often fails to account for the functional significance of coronary lesions. The pathophysiology of acute coronary syndromes reveals that many events are driven not by overtly obstructive plaques, but by vulnerable lesions or supply–demand mismatches, which are not visible to angiography alone. As such, a strategy that integrates functional assessment into revascularization decisions is critical. Fractional flow reserve, introduced to address this gap, demonstrated clear clinical benefits in landmark trials such as FAME and FAME2 [23,24]. However, its clinical uptake has been limited by practical challenges, including procedural complexity, the need for hyperemic agents, and patient discomfort. To overcome these barriers, iFR was developed. Like FFR, iFR is wire-based but does not require hyperemia; it measures the pressure gradient during a specific diastolic period when microvascular resistance is naturally minimized. The American College of Cardiology, American Heart Association, and Society for Cardiovascular Angiography and Interventions recommend FFR or iFR to guide PCI in patients with intermediate lesions, as both reduce unnecessary stenting and improve outcomes compared to angiography alone [25,26]. Notably, iFR offers the benefits of shorter procedure times and avoids adenosine-related side effects, while being non-inferior to FFR regarding MACEs at 12 months after PCI [6,7]. However, iFR still requires a wire-based technique during the procedure.

Recently, quantitative flow ratio, an angiography-derived physiological index that computationally simulates FFR without requiring pressure wires or pharmacological hyperemia, represents a promising solution [27]. QFR holds immense potential to transform coronary physiology from a specialized technique into a routine component of PCI decision-making. Its non-invasive, cost-effective, and workflow-friendly nature makes it particularly appealing in healthcare systems with limited resources or where FFR utilization is low. QFR can be completed in under five minutes in common practice: first, operators acquire two high-quality angiographic projections of the target vessel (ideally 25–45° apart, free of overlap or foreshortening, recorded at ≥15 frames per second and during end-diastole to minimize motion artifact), after which a dedicated software performs 3D quantitative coronary angiography to delineate the vessel lumen and mark proximal and distal reference points, estimates flow velocity (typically via the TIMI frame-count method, now often automated), and integrates these data to calculate the distal/proximal pressure ratio, with ≤0.80 signifying hemodynamically significant stenosis. The operator then verifies the 3D reconstruction and, if needed, repeats the analysis with alternative views to ensure accuracy [10,12,28,29,30,31,32]. Because only stored angiograms are required, QFR can be obtained remotely and retrospectively whenever at least two suitable angiographic projections of the target vessel are captured during PCI. These advantages are particularly beneficial for patients with ACS who also have non-culprit lesions. After treating the culprit lesion, operators can obtain QFR-standardized images of the non-culprit vessels and send them for off-site QFR analysis in cases where the hospital lacks dedicated QFR software. This “capture-and-send” strategy enables accurate physiological assessment and decision-making without requiring additional equipment or in-lab expertise, offering significant practice implications for underserved environments. Multiple studies, including FAVOR II China and FAVOR II Europe-Japan, have demonstrated that QFR outperforms visual estimation in identifying ischemia-producing lesions and enhances diagnostic accuracy across lesion complexities [12,33]. In a systematic review and Bayesian meta-analysis, Collet et al. demonstrated that QFR outperformed angiography across a range of lesion complexities, with significantly improved sensitivity and specificity for identifying physiologically significant stenosis [34]. Notably, another angiography-derived, wire-free, and adenosine-free method known as vessel fractional flow reserve (vFFR), which uses different algorithms and software platforms compared to QFR, is also currently undergoing validation in clinical trials [28,35,36]. While vFFR demonstrates potential advantages similar to QFR, it falls beyond the scope of the current study and will be examined separately.

Building upon this diagnostic validation, clinical trials have begun to explore whether QFR guidance translates into improved patient outcomes. The FAVOR III China trial, the first large-scale randomized controlled trial (RCT) in this domain, demonstrated a significant reduction in major adverse cardiovascular events (MACEs) at one year with QFR-guided PCI compared to angiography-guided PCI (18.5% vs. 24.4%; HR 0.71; *p* = 0.004), primarily driven by fewer myocardial infarctions and ischemia-driven revascularizations [17]. Notably, these benefits persisted at two-year follow-up. In our meta-analysis, which pooled data from three RCTs (Ullrich-Daub et al., Barauskas et al., and FAVOR III China), QFR-guided PCI showed non-significant but consistently favorable trends in reducing MACEs, repeat revascularizations, and rehospitalizations at one year [15,16,17]. However, substantial heterogeneity was observed for MACEs (I^2^ = 84.95%) and revascularization (I^2^ = 94.18%), indicating variability in study populations, endpoints, and methodologies.

Differences in patient profiles and study designs may partly explain the variation in results. A trial carried out by Ullrich-Daub et al. [15], conducted in ACS patients with multivessel disease, did not find significant differences in outcomes between QFR- and angiography-guided arms. Their endpoint incorporated subjective components, such as angina scores, which potentially diluted the signal of hard clinical events [15]. In contrast, Barauskas et al. focused exclusively on STEMI patients and observed significant reductions in mortality and revascularization with QFR guidance, supporting the notion that QFR may be especially beneficial in high-risk populations [16].

Similarly, the FIRE trial demonstrated the predictive utility of QFR in older MI patients with multivessel disease. The study randomized 1445 older MI patients to either a culprit-only revascularization strategy or a physiology-guided complete revascularization strategy for non-culprit lesions. In the physiology-guided complete revascularization group, QFR and wire-based physiology were compared for non-culprit vessels, with QFR used in 35.2% of cases. No significant difference was found in vessel-oriented composite endpoints (hazard ratio 0.57, 95% CI 0.28–1.15). In the culprit-lesion only group, 40.5% of non-culprit lesions had a QFR value ≤ 0.8, and low QFR values were significantly associated with higher vessel-oriented composite endpoints (22.1%) compared to normal QFR values (7.1%) (*p* < 0.001) [37,38]. Furthermore, the ongoing FAVOR III Europe-Japan trial (NCT03729739) aims to compare QFR with FFR across 2000 patients and will provide vital insight into long-term outcomes and applicability in Western populations [39].

The AQVA trial demonstrated that QFR-based virtual PCI significantly improved post-PCI optimal physiological results (QFR ≥ 0.90), which was attributed to the angiography-based group underestimating a diseased segment outside the stented one [40]. Additional insights are provided by the HAWKEYE study, which found that suboptimal post-PCI QFR values (≤0.89) were associated with a nearly threefold increase in vessel-related adverse events, specifically vessel-oriented composite endpoints including vessel-related cardiovascular death, vessel-related myocardial infarction, and ischemia-driven target vessel revascularization, even after angiographically successful PCI [41]. Indeed, QFR allows clinicians to prospectively plan, execute, and validate interventions based on a continuous, quantitative, and patient-specific physiological metric.

Despite these advantages, limitations remain. QFR requires high-quality angiographic images with adequate orthogonal views, as suboptimal image acquisition, such as poor contrast, vessel overlap, foreshortening, or inadequate separation, can compromise its accuracy and feasibility. Inadequate images may lead to exclusion of vessels or unreliable results, as seen in the FAVOR III China trial [17,42]. Additionally, QFR accuracy is reduced in certain lesion subsets, such as bifurcation lesions, diffuse disease, tandem lesions, ostial lesions, and chronic total occlusions, which affect the computational assumptions and lower diagnostic performance, particularly in borderline FFR zones and settings involving acute myocardial infarction [43]. Furthermore, QFR does not directly measure coronary microvascular dysfunction (CMD), and its diagnostic performance may be influenced by the presence of microvascular dysfunction [44]. However, recent studies utilizing various indices such as contrast-flow QFR, fixed-flow QFR, and hyperemic flow velocity to predict CMD have demonstrated promising results [45,46]. Operator experience and lack of standardized QFR protocols can also influence outcomes [17,42,47]. The accuracy and reproducibility of QFR depend on strict adherence to protocols, and variability in operator expertise can introduce differences that impact clinical decision-making.

Ultimately, this meta-analysis is limited by the inclusion of only three randomized controlled trials, which potentially restricts generalizability, reduces statistical power, and increases the risk of type II error. Heterogeneity across these studies may result from variations in patient populations, endpoint definitions, and clinical protocols, which could influence the interpretation of this meta-analysis. Furthermore, the lack of a cost-effectiveness analysis and the limited follow-up duration of one year constrain the evaluation of economic and long-term impacts.

## 5. Conclusions

Quantitative Flow Ratio (QFR), a non-invasive imaging-based tool, has demonstrated comparable one-year clinical outcomes to traditional angiography-guided PCI. Across multiple studies, QFR-guided interventions showed numerically lower but statistically insignificant risks of MACEs, revascularization, and rehospitalization. While these findings suggest feasibility and comparable safety, larger high-quality randomized controlled trials with longer follow-up are needed to confirm any clinical benefits and further define QFR’s role in routine clinical practice.

## Figures and Tables

**Table 1:** Baseline characteristics of included studies.

Study	Design	Country	Population	Sample Size	Outcomes	MACEs	Follow-Up Duration
**Ullrich-Daub, 2024** [15]	Randomized, 2-center, superiority trial	Germany	ACS with multivessel disease (STEMI/NSTEMI/UA)	202	Death, non-fatal MI, unplanned hospitalization for angina/HF, unplanned revascularization, stroke, SAQ < 90	Death, unplanned PCI, stroke	12 months
**Barauskas, 2023** [16]	Randomized, single-center, prospective trial	Lithuania	STEMI with ≥1 non-culprit intermediate lesion	198	Mortality, revascularization, rehospitalization, physical activity limitations	Death, culprit/non-culprit coronary artery revascularization	12 months
**Xu, 2021** [17]	Randomized, multicenter, sham-controlled trial	China	CAD with 50–90% stenosis, stable/unstable angina, or post-MI > 72 h	3825	Death, MI, ischemia-driven revascularization	Death, MI, ischemia-driven revascularization	12 months

SAQ: Seattle Angina Questionnaire; MI: myocardial infarction; CAD: coronary artery disease.

## Data Availability

All derived data supporting the findings of this study are available from the corresponding author upon reasonable request.

## Abbreviations

ACS	Acute Coronary Syndrome
CAD	Coronary Artery Disease
CMD	Coronary Microvascular Dysfunction
FFR	Fractional Flow Reserve
iFR	Instantaneous Wave-Free Ratio
IHD	Ischemic Heart Disease
MACE	Major Adverse Cardiovascular Events
MI	Myocardial Infarction
PCI	Percutaneous Coronary Intervention
QFR	Quantitative Flow Ratio
vFFR	Vessel Fraction Flow Reserve
